# A New Strategy for the Synthesis of Fluorinated Polyurethane

**DOI:** 10.3390/polym11091440

**Published:** 2019-09-02

**Authors:** Pu-Cheng Wang, Dan Lu, Hu Wang, Ru-Ke Bai

**Affiliations:** 1 Institute of Nuclear Physics and Chemistry, China Academy of Engineering Physics, Mianyang 621000, China; 2 CAS Key Laboratory of Soft Matter Chemistry, Department of Polymer Science and Engineering, University of Science and Technology of China, Hefei 230026, China

**Keywords:** fluorinated polyurethane, fluorinated polymer, chlorotrifluoroethylene

## Abstract

An alternating fluorinated copolymer based on chlorotrifluoroethylene (CTFE) and butyl vinyl ether (BVE) was synthesized by RAFT/MADIX living/controlled polymerization in the presence of S-benzyl O-ethyl dithiocarbonate (BEDTC). Then, using the obtained poly(CTFE-alt-BVE) as a macro chain transfer agent (macro-CTA), a block copolymer was prepared by chain extension polymerization of vinyl acetate (VAc). After a basic methanolysis process, the poly(vinyl acetate) (PVAc) block was transferred into poly(vinyl alcohol) (PVA). Finally, a novel fluorinated polyurethane with good surface properties due to the mobility of the flexible fluorinated polymer chains linked to the network was obtained via reaction of the copolymer bearing the blocks of PVA with isophorone diisocyanate (IPDI) as a cross-linking agent.

## 1. Introduction

Fluorinated polyurethanes, a new class of functional materials, combine some virtues of fluorinated polymer and polyurethane, such as high thermal stability, good mechanical properties, excellent chemical resistance (to acids, alkalis, and solvents), and attractive surface properties. [1,2,3,4,5,6,7,8,9,10,11,12,13,14,15,16] As a result, they have attracted considerable interest in recent years.

Fluorocarbon chains or groups have been incorporated into polyurethanes by fluorinated chain extenders, [17,18,19,20,21] diisocyanates, [22] glycols, [23,24,25,26,27,28,29,30,31,32,33,34,35,36,37,38,39,40,41] side groups/chains [42,43,44,45,46,47,48,49,50,51,52,53,54,55,56,57,58], and end-cappers [59,60,61,62,63,64,65,66]. In 2005, for the first time, a novel fluorinated polyurethane based on polyurethane macromonomers partly acrylate-endcapped with hexafluorobutyl acrylate was synthesized through macromonomer radical copolymerization [17]. Synthesis of fluorinated polyurethane with fluorinated diisocyanates the less used due to the high synthesizing cost and poor variability of fluorinated diisocyanate. Takakura et al. produced a series of fluorine-containing poly(urethane-urea)s with 2,2,3,3,4,4,5,5-octafluorohexamethylene diisocyanate in 1990 [22]. Fluorinated polyurethanes synthesized with fluorinated diols as soft segments are more common. In 1992, Ho prepared a fluorinated polyurethane based on ethylene-fluoroalkyl-ethylene diol 3-(trifluoromethyl)-3,4,4,5,5,6,6,7,7,8,8-undecafluoro-l,10-decanediol and 1,6-hexamethylene diisocyanate [40]. Liu et al. prepared a series of main-chain-fluorinated thermoplastic polyurethane elastomers based on fluoropoly(oxyalky1ene)diol by one-step bulk polymerization [41]. Introducing fluorinated side groups/chains is another common method for the preparation of fluorinated polyurethanes. In 1995, Chapman et al. reported the synthesis and characterization of a series of fluorinated polyurethanes with fluorinated side groups (–CH_2_(CF_2_)_n_CF_3_, n = 0, 2, 3, 6) on the soft blocks [58]. Recently, Shi et al. synthesized several fluorinated polyurethanes containing fluoroether side groups on the hard segments [54]. Fluorinated polyurethanes prepared by fluorinated alcohols as end-cappers usually show no evident improvement in properties compared with hydrogenated polyurethanes, owing to the less fluorine content and lower molecular weight of fluorinated alcohols. Although many strategies for the synthesis of fluorinated polyurethanes have been proposed, it is still a challenge to introduce fluorinated long chains with defined length or molar mass into polyurethanes.

As we know, living/controlled radical polymerization is a powerful tool for the synthesis of well-defined polymers with predetermined molar mass, low dispersity, and various architectures [67,68,69,70]. Some successful works focusing on the living/controlled radical (co)polymerization for fluoroolefins, such as vinylidene fluoride (VDF), tetrafluoroethylene (TFE), 3,3,3-trifluoropropene (TFP), hexafluoropropylene (HFP), and chlorotrifluoroethylene (CTFE), have been done in recent years [71,72,73,74,75,76,77,78,79,80,81]. RAFT/MADIX polymerization initiated by γ-rays seems to be very suitable for fluoroolefins. In 2011, Liu et al. successfully achieved RAFT/MADIX copolymerization of CTFE and butyl vinyl ether (BVE) at room temperature under ^60^Co γ-ray irradiation with a xanthate as a mediating agent [75]. Another fluorinated copolymer, poly(HFP-alt-BVE), was prepared under similar conditions in 2013 [77]. Later in 2014, RAFT/MADIX copolymerization of CTFE and N-vinylpyrrolidone under ^60^Co γ-ray irradiation with S–benzyl O–ethyl dithiocarbonate (BEDTC) as the mediating agent was reported [78]. However, there is a problem that could not be ignored in the polymerization reactions irradiated by ^60^Co γ-ray. In fact, the hyper-powerful energy of γ-rays could cause many unexpected and complicated crosslinking reactions during the polymerization process.

**Scheme 1:**
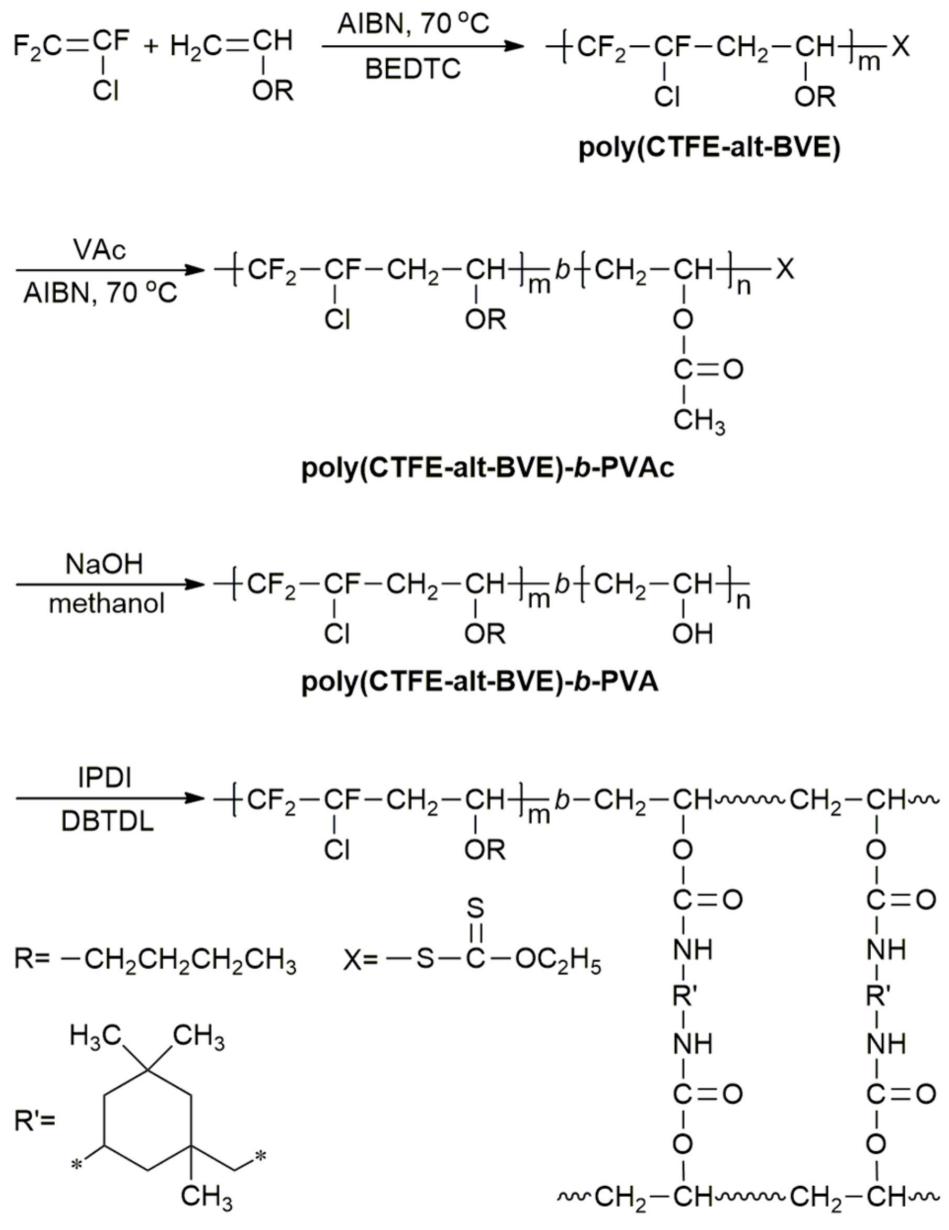
Schematic representation of the synthetic procedures of fluorinated polyurethane. AIBN: azodiisobutyronitrile, BEDTC: S–benzyl O–ethyl dithiocarbonate, CTFE: chlorotrifluoroethylene, BVE: butyl vinyl ether, VAc: vinyl acetate, PVAc: poly(vinyl acetate), PVA: poly(vinyl alcohol), IPDI: isophorone diisocyanate, DBTDL: dibutyltin dilaurate.

In this work, we firstly prepared a fluorinated copolymer (poly(CTFE-alt-BVE)) with defined molar mass and low dispersity by RAFT/MADIX copolymerization of CTFE and BVE using BEDTC as a chain transfer agent in moderate conditions without ^60^Co γ-rays in order to avoid the unexpected crosslinking reactions. Based on this, we successfully prepared a novel crosslinked fluorinated polyurethane with flexible fluorinated polymer chains linked to the network. As the fluorinated polymer chains can easily migrate to the surface of the matrix, the surface properties of the material were significantly improved. The whole reaction process is depicted in Scheme 1.

## 2. Materials and Methods 

### 2.1. Materials

CTFE was purchased from Zhejiang Juhua Co., Ltd, Quzhou, China. BVE was obtained from Hubei Xinjing New Materials Co., Ltd, Wuhan, China, was dried with CaH_2_ and distilled under reduced pressure before use. VAc, ethyl acetate, methanol, tetrahydrofuran (THF), and NaOH were purchased from Sinopharm Chemical Reagent Co., Ltd (Shanghai, China). VAc was purified by passing through a basic alumina column, and subsequently distilled. Ethyl acetate was refluxed and distilled over CaH_2_. AIBN, isophorone diisocyanate (IPDI), and dibutyltin dilaurate (DBTDL) were purchased from Aladdin Industrial Corporation (Shanghai, China). AIBN was recrystallized from methanol twice before use. BEDTC was synthesized according to a previous report [82] (As shown in the Supplementary Section 1). All other chemicals were used as received unless otherwise noted.

### 2.2. Characterization

^1^H, ^19^F, and ^13^C NMR spectra were recorded on a Bruker DPX-400 spectrometer (Bruker Corporation, Billerica, USA), using CDCl_3_ as a solvent. All NMR tests were carried out at 25 °C.

The values of the number-average molar mass (*M_n_*) and dispersity (*M_w_*/*M_n_*) were determined by means of a Waters 150C gel permeation system (Waters Corporation, Milford, USA) equipped with 10^3^, 10^4^, and 10^5^ Å Waters Ultrastyragel columns and a light scattering detector, using THF (1.0 mL·min^−1^) as the eluent at 25 °C, and the calibration was carried out with polystyrene standards.

FT-IR spectra were recorded on a Bruker VECTOR-22 infrared spectrometer (Bruker Corporation, Billerica, USA).

X-ray photoelectron spectroscopy (XPS) analysis was performed on a Thermo-VG Scientific ESCALab 250 instrument (Thermo Fisher Scientific Inc, Waltham, USA) with Al Kα radiation as the excitation source.

Static contact angles were measured with an optical contact angle meter (Solon (Shanghai) technology science Co. Ltd.) at ambient temperature.

Thermogravimetric analysis (TGA) was performed with a DTG-60H apparatus from Shimadzu (Kyoto, Japan) at a heating rate of 10 °C·min^−1^ from room temperature up to a maximum of 700 °C under nitrogen.

Chemical resistance was evaluated by spot test according to ASTM D1308. The dried coating with thickness of about 10 μm was exposed to droplets of different solvents for 24 h at room temperature in a closed box kept in equilibrium with the solvent vapor. The droplets were then wiped, and the chemical resistance was determined by evaluating the changes in coating integrity, haze, or hardness. A visual scale was used to compare these changes: 10 (no effect), 8 (light haze), 6 (softening), 4 (cracks), 2 (heavily damaged), 0 (completely solubilized).

### 2.3. Synthetic Procedures

#### 2.3.1. General Procedure for RAFT/MADIX Copolymerization of CTFE and BVE

The reaction was performed in a 30 mL stainless steel autoclave equipped with a manometer, a safety inlet valve, and a magnetic stirrer. First, a mixture of 5.1 mg AIBN (0.031 mmol), 33.5 mg BEDTC (0.158 mmol), 2.0 mL BVE (15.8 mmol), and 5.0 mL ethyl acetate was introduced into the autoclave. Then, the vessel was closed and immersed in liquid nitrogen for 15 min. After several nitrogen–vacuum cycles to remove any trace of oxygen, 2.5 g CTFE (21 mmol) was condensed into the autoclave via a mass flow meter, and its exact amount was assessed by double weighing (weight difference before and after filling the autoclave with CTFE). The polymerization was carried out at 70 °C for a predetermined time and then quenched by cooling with ice water. The unreacted CTFE was slowly vented. The solution was concentrated and precipitated in methanol. The polymer was finally dried under vacuum at 50 °C until a constant weight was attained, and the conversion of the monomer was determined by gravimetry. Preparation of items for XPS, chemical resistance and static contact angle measurements: 0.2 g of the obtained copolymer was dissolved in 1.0 mL ethyl acetate, and then the solution was cast onto glass plates, followed by drying at 70 °C for 12 h.

#### 2.3.2. Synthesis of Poly(CTFE-alt-BVE)-*b*-PVAc

Poly(CTFE-alt-BVE) (0.783 g) bearing a xanthate end-group as a macro chain transfer agent (macro-CTA) was dissolved in 5 mL ethyl acetate and charged in a 10 mL glass tube with 3.0 mg AIBN (0.018 mmol) as well as 1.5 mL VAc (16.2 mmol). Three cycles of freeze-vacuum-thaw were conducted to remove any traces of oxygen. After sealing under vacuum, the tube was immersed in a thermostatic oil bath at 70 °C for 6 h. Then the tube was cooled down by ice water to stop the polymerization, and the solution was precipitated into methanol. Purification was carried out by repeating dissolution in ethyl acetate and precipitation from methanol. The resultant polymer was collected and dried under vacuum at 50 °C until a constant weight. The conversion of VAc was determined by gravimetry.

#### 2.3.3. Synthesis of Poly(CTFE-alt-BVE)-*b*-PVA

A mixture of 0.806 g poly(CTFE-alt-BVE)-*b*-PVAc in 20 mL THF and 0.200 g NaOH (5.00 mmol) in 20 mL methanol was introduced into a 100 mL round-bottom flask equipped with a condenser and a magnetic stirrer. Then it was immersed in an oil bath at 40 °C and stirred for 6 h. After cooling to room temperature, the solution was concentrated and precipitated in methanol. A similar purification process as the one descripted above was conducted by repeating dissolution in THF and precipitation from methanol in order to remove the residual base and the salt produced during the reaction. The resultant block polymer was dried under vacuum at 50 °C until a constant weight.

#### 2.3.4. Preparation of the Fluorinated Polyurethane Coating

A mixture of poly(CTFE-alt-BVE)-*b*-PVA (0.56 g), IPDI (0.10 g, 0.45 mmol), and DBTDL (0.56 mg, 8.9 × 10^−4^ mmol, added by taking 100 μL dilute solution of DBTDL in THF with a concentration of 5.6 mg·cm^−3^) in 10 mL THF was charged in a 25 mL round-bottom flask equipped with a condenser and a magnetic stirrer. The solution was stirred at 65 °C for 10 h. The fluorinated polyurethane coating was prepared by casting the viscous solution onto glass plates, drying at 70 °C for 12 h, and further drying at 80 °C for 12 h in a vacuum oven to remove any residual solvent and enhance the crosslinking reaction.

## 3. Results and Discussion

Table 1 shows the results of RAFT/MADIX copolymerization of CTFE and BVE. The theoretical molar masses (*M_n,th_*) and the molar masses determined by ^1^H NMR spectra (*M_n,NMR_*) of poly(CTFE-alt-BVE) were respectively calculated according to Equation (1) and (2):

*M_n,th_* = ([BVE]_0_/[BEDTC]_0_) × *Conv.* × (*M_CTFE_* + *M_BVE_*) + *M_BEDTC_* (1)

*M_n,NMR_* = (*I*_3.76_/*I*_7.35_) × 2.5 × (*M_CTFE_* + *M_BVE_*) + *M_BEDTC_* (2)

where [BVE]_0_/[BEDTC]_0_ is the initial molar ratio of BVE to BEDTC, *Conv.* is the conversion of be, *M_CTFE_*_,_ *M_BVE_*_,_ and *M_BEDTC_* stand for the molar mass of CTFE, be, and BEDTC, respectively, *I*_3.76_ and *I*_7.35_ are the integral values of ^1^H NMR signal peaks of poly(CTFE-alt-BVE) centered at 3.76 and 7.35 ppm, which belong to the side butoxyl groups (–OC***H_2_***CH_2_CH_2_CH_3_) and the phenyl end groups of BEDTC, respectively.

As shown in Table 1, the molar masses of poly(CTFE-alt-BVE) calculated from the ^1^H NMR spectra are close to the theoretical values. Although the molar masses determined by GPC (*M_n,GPC_*) were systematically higher than the theoretical values because of the polystyrene standards used in the calibration, the dispersities were narrow.

**Figure 1:**
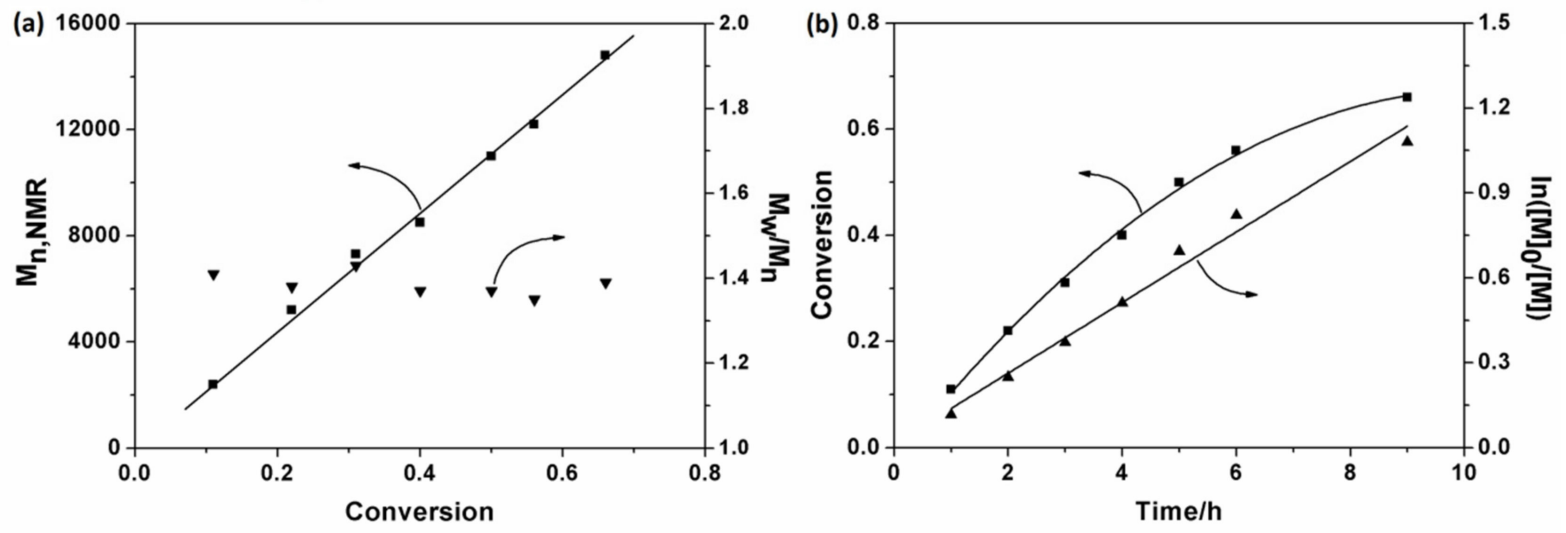
Kinetics of the copolymerization of CTFE and BVE: (**a**) molar mass and dispersity as functions of monomer conversion; (**b**) monomer conversion and ln([M]_0_/[M]) as a function of polymerization time.

Figure 1a reveals the evolution of the molar mass and dispersity as functions of monomer conversion during copolymerization of CTFE and BVE. It can be observed that the molar mass of poly(CTFE-alt-BVE) increased linearly with the monomer conversion, and the dispersity values remained small. Moreover, a linear relationship between ln([M]_0_/[M]) and the polymerization time is shown in Figure 1b, which indicates that the polymerization was a first-order reaction with respect to BVE concentration, and the number of active radicals remained constant during the polymerization [70]. All these results demonstrate that a living/controlled copolymerization of CTFE and BVE took place under the chosen reaction condition.

**Figure 2:**
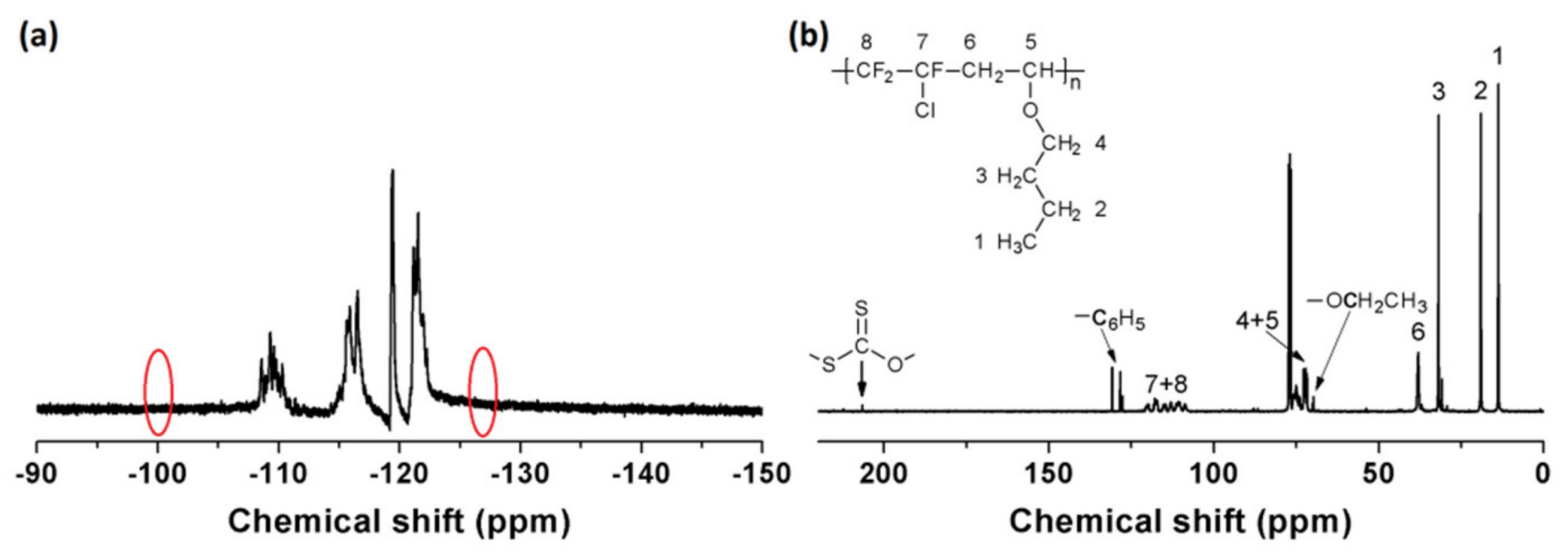
(**a**) ^19^F and (**b**) ^13^C NMR spectra of poly(CTFE-alt-BVE) (Sample P4).

The chemical structure of the obtained copolymer was characterized by ^19^F, ^13^C, and ^1^H NMR spectroscopy, and the sample P4 was chosen as a representative sample. It showsed a series of multiplets centered at −109.4, −116.8, −119.6, and −121.6 ppm in the ^19^F NMR spectrum (Figure 2a), which can be related to the constitutive CF_2_ and CFCl groups of the chain backbone [83]. Furthermore, the absence of peaks at −100 ppm [84] and −127 ppm [85] in ^19^F NMR spectrum indicates that there were no CTFE–CTFE diads in the polymer chains, whi ch is a remarkable evidence of the alternating structure of poly(CTFE-alt-BVE), given that BVE cannot homopolymerize at those radical polymerization conditions. Figure 2b shows the ^13^C NMR spectrum of poly(CTFE-alt-BVE). The signal around 70.0 ppm and the weak signal around 212.3 ppm were ascribed to the methylene linked to the oxygen atom and the quaternary carbon in the structure of –S–***C***(=S)–O–H_2_-CH_3_ at the chain end of the polymer, respectively. The signals located between 127 and 131 ppm were assigned to the carbon atoms of phenyl groups.

**Figure 3:**
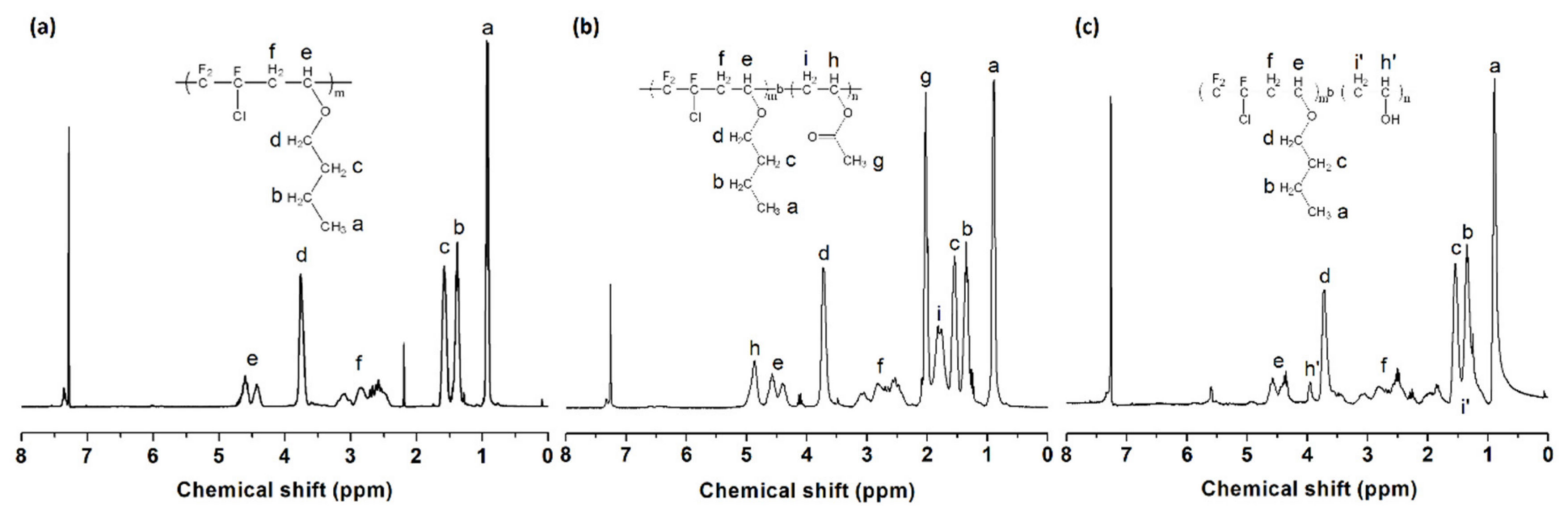
^1^H NMR spectra of (**a**) poly(CTFE-alt-BVE) (Sample P4), (**b**) poly(CTFE-alt-BVE)-*b*-PVAc, and (**c**) poly(CTFE-alt-BVE)-*b*-PVA.

Figure 3a shows the ^1^H NMR spectrum of poly(CTFE-alt-BVE). The resonances centered at 4.60 and 4.41 ppm were attributed to the protons of methine in the structure of –CTFE–CH_2_–C***H***(OBu)–CTFE–, and the split arose from the chiral carbons in the polymer chain. The broad peaks located at 2.40–3.30 ppm were ascribed to the methylene of the BVE units in the polymer backbone (Supplementary Section 2), and the resonances at 3.76, 1.58, 1.38, and 0.93 ppm to the side butoxyl groups. In addition, the peaks at 7.35 ppm belonged to the phenyl end groups of BEDTC (As shown in Figures S1 and S2), indicating that a chain transfer reaction occurred. The signals of the ethoxyl groups overlapped with those of the polymer backbone.

**Figure 4:**
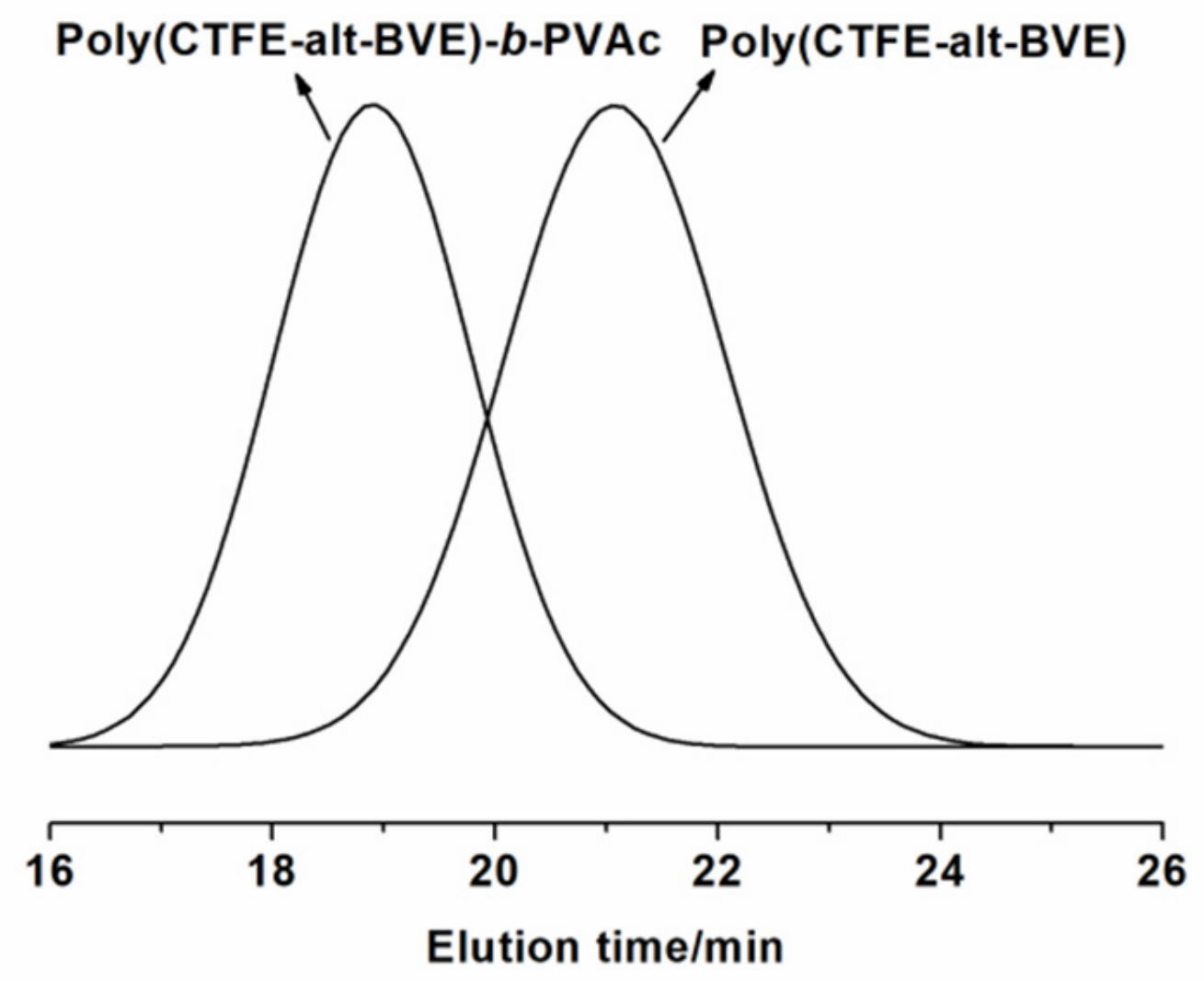
GPC traces of poly(CTFE-alt-BVE) (Sample P4, *M_n,GPC_* = 10900, *M_w_*/*M_n_* = 1.37) and poly(CTFE-alt-BVE)-*b*-PVAc (*M_n,GPC_* = 17200, *M_w_*/*M_n_* = 1.32).e.

Chain extension polymerization was performed using Sample P4 as a macro-CTA and VAc as the monomer. Figure 4 shows the GPC traces of the obtained block copolymer (poly(CTFE-alt-BVE)-*b*-PVAc) and the macro-CTA. A symmetrical and monomodal curve for the block copolymer with a shift toward a higher molecular weight related to the curve of the macro-CTA in the GPC traces indicates that the chain extension polymerization was successfully achieved and the dispersity of the block copolymer was still low.

**Figure 5:**
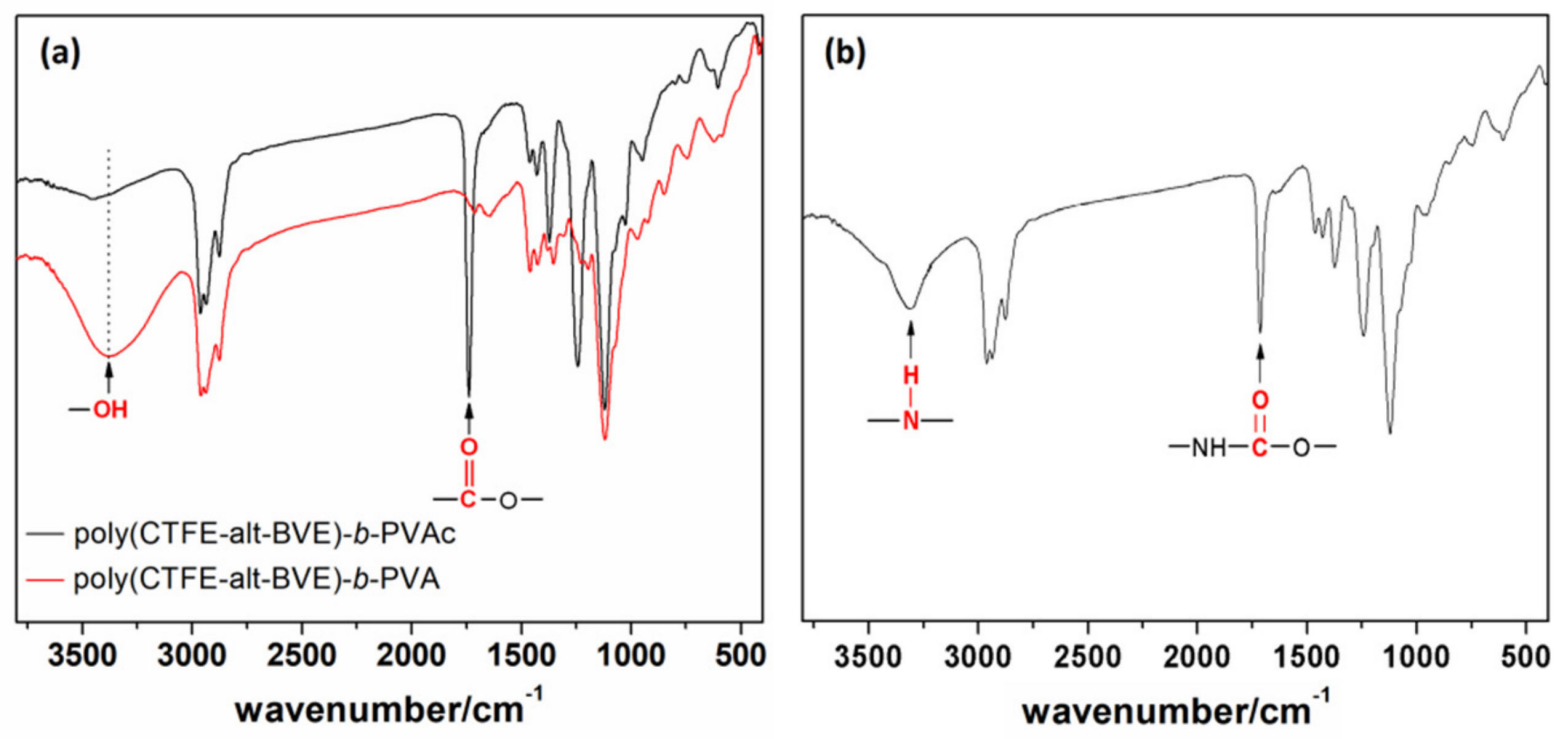
FT-IR spectra of (**a**) poly(CTFE-alt-BVE)-*b*-PVAc, poly(CTFE-alt-BVE)-*b*-PVA, and (**b**) the fluorinated polyurethane.

The obtained poly(CTFE-alt-BVE)-*b*-PVAc was characterized by ^1^H NMR spectroscopy (Figure 3b). Some new signal peaks appeared compared to the spectrum of poly(CTFE-alt-BVE). The resonance signals centered at 1.79 and 4.87 ppm can be attributed to the protons of the methene and methine groups in the VAc unit, respectively. A single peak signal appeared at 2.02 ppm, which belongs to the methyl groups of the VAc units. The block copolymer, poly(CTFE-alt-BVE)-*b*-PVAc, was then hydrolyzed in basic methanol, and the resulting amphiphilic block copolymer, poly(CTFE-alt-BVE)-*b*-PVA, was confirmed by ^1^H NMR and FT-IR spectroscopy. Figure 3c shows the ^1^H NMR spectrum of poly(CTFE-alt-BVE)-*b*-PVA. As can be seen, the signal peaks attributed to the VAc unit almost disappeared, and new resonance signals belonging to the vinyl alcohol (VA) unit were recorded. The resonance signal centered at 3.95 ppm belongs to the methine groups in the VA unit, while the resonance signal of the methene group in the VA unit overlaps with that of the butoxyl group in the BVE unit. Figure 5a shows the FT-IR spectra of poly(CTFE-alt-BVE)-*b*-PVAc and poly(CTFE-alt-BVE)-*b*-PVA. The strong absorption band of the C=O bond at 1740 cm^−1^ in the VAc unit almost completely disappeared after hydrolysis, and a new band at 3378 cm^−1^ appeared, which could be attributed to stretching vibration of the O–H bond in the VA unit.

Figure 5b shows the FT-IR spectrum of the fluorinated polyurethane. As can be seen, characteristic peaks of the N–H bond at 3305 cm^−1^ and of the C=O bond at 1715 cm^−1^ clearly appeared in the spectrum, which demonstrates the successful preparation of the fluorinated polyurethane [38,39].

**Figure 6:**
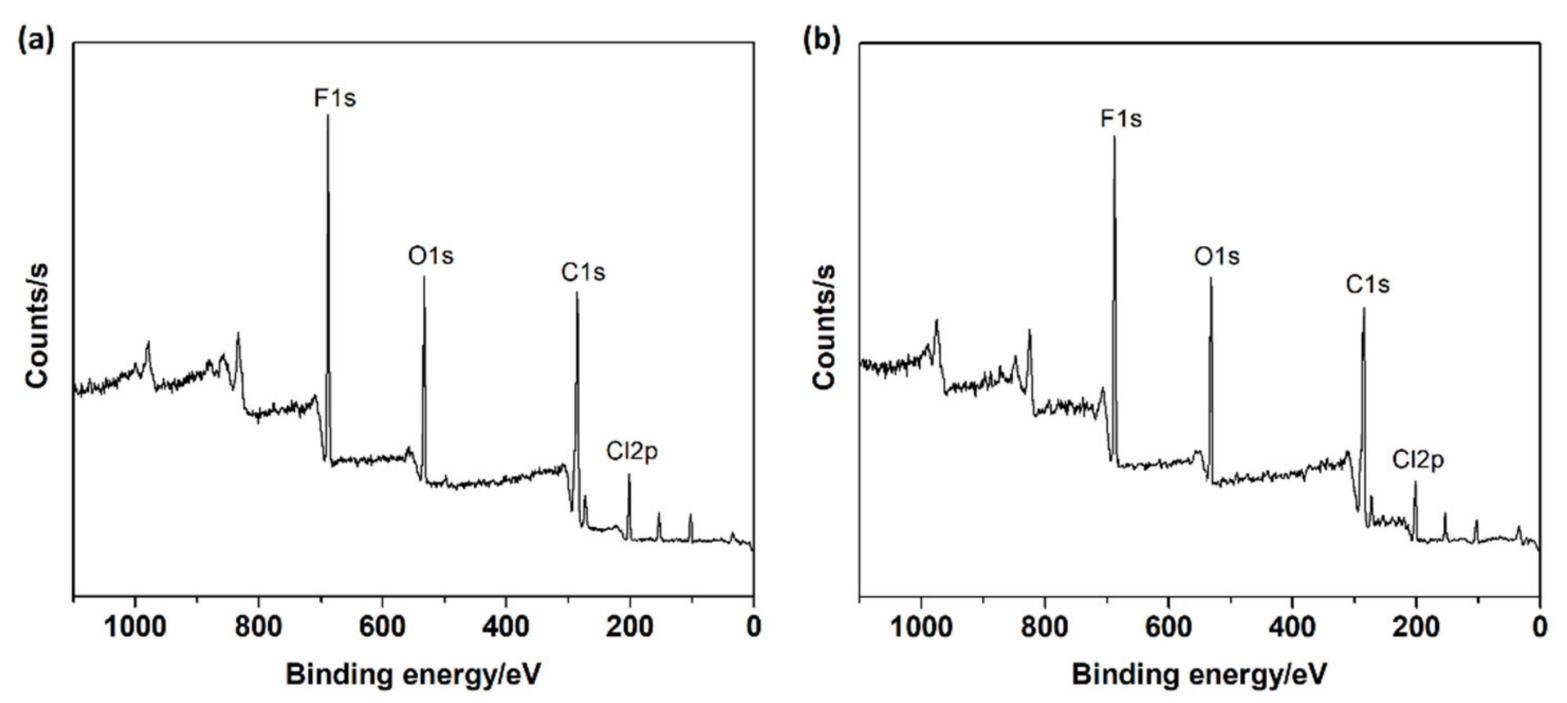
X-ray photoelectron spectroscopy (XPS) survey spectra of (**a**) the fluorinated polyurethane coating and (**b**) poly(CTFE-alt-BVE) coating (Sample P4).

The surface composition of the fluorinated polyurethane coating was studied by XPS analysis (Figure 6a). The XPS measurement showed the photoionization peaks of chlorine, carbon, oxygen, and fluorine in the survey spectrum, but no peak attributed to nitrogen was detected. In fact, the XPS spectrum of the fluorinated polyurethane was very similar to that of poly(CTFE-alt-BVE) (Figure 6b). This was probably due to the low free energy of fluorine and the mobility of the flexible fluorinated polymer chains linked to the coating network, which led the poly(CTFE-alt-BVE) blocks to the outermost surface of the coating [54].

**Figure 7:**
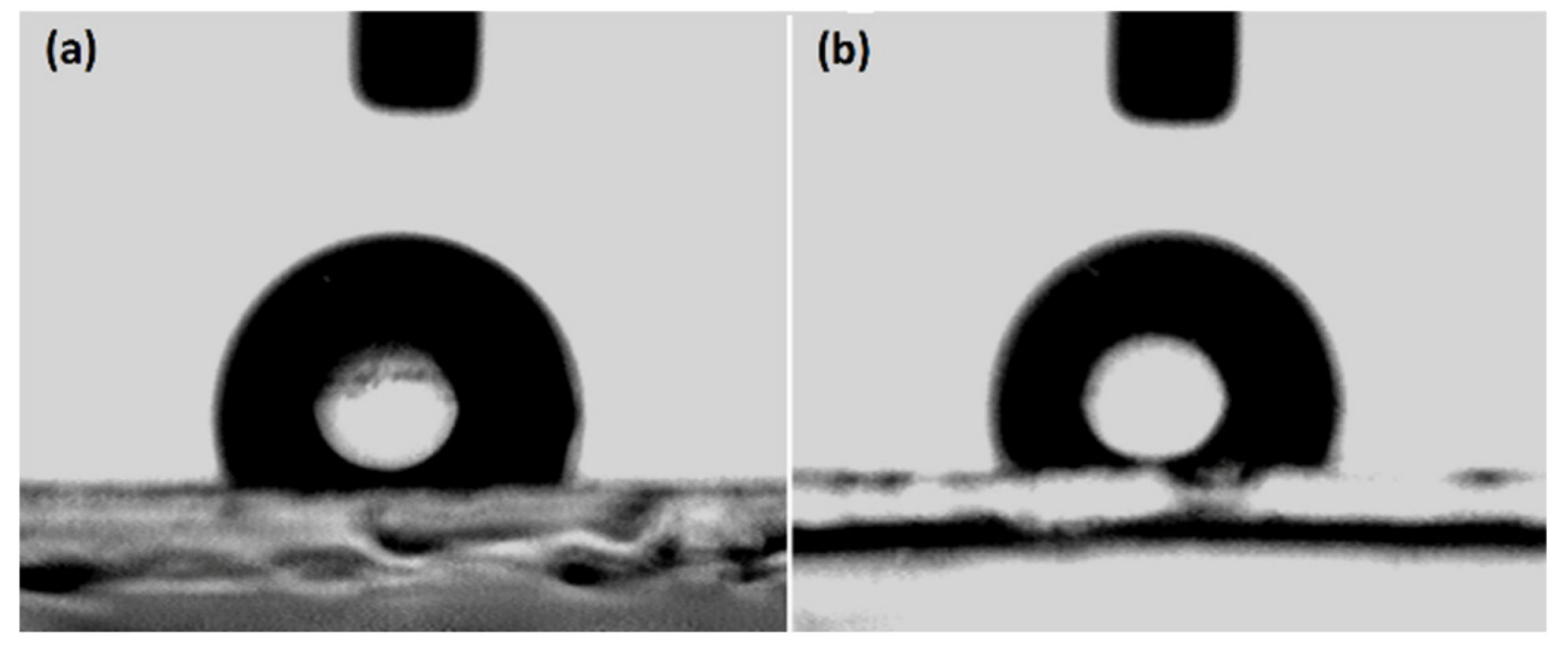
Photographs taken during static water contact angle measurements: (**a**) fluorinated polyurethane coating, (**b**) poly(CTFE-alt-BVE) coating (Sample P4).

The surface properties of the fluorinated polyurethane coating were evaluated by static contact angle measurements (Figure 7). The water contact angle of the fluorinated polyurethane coating was 108°, which is equal to the value of the viscous coating made of poly(CTFE-alt-BVE). This results was consistent with the above-mentioned XPS analysis result.

**Figure 8:**
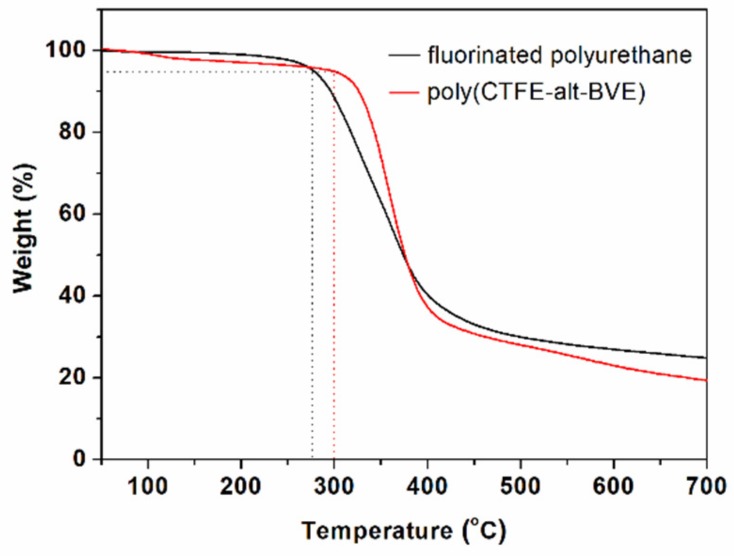
TGA thermograms of the fluorinated polyurethane (black) and of poly(CTFE-alt-BVE) (red, Sample P4).

The thermostability of the fluorinated polyurethane was characterized by TGA (Figure 8). According to the TGA thermogram, the decomposition temperature of the fluorinated polyurethane at 5% weight loss was about 277 °C, which demonstrates that the fluorinated polyurethane prepared in this work had good thermal stability, although it was a bit lower than that of poly(CTFE-alt-BVE).

The prepared fluorinated polyurethane coating is not any more viscous than the initial poly(CTFE-alt-BVE) and is insoluble in common organic solvents, such as ethyl acetate, acetone, CHCl_3_, and THF, which improves the applicability of this material. Table 2 shows the chemical resistance of the fluorinated polyurethane and of poly(CTFE-alt-BVE). As can be seen, the fluorinated polyurethane has an excellent chemical resistance due to the presence of fluorinated chains and a crosslinking structure.

## 4. Conclusions

Living/controlled radical copolymerization of CTFE and BVE has been successfully achieved using BEDTC as a chain transfer agent and AIBN as an initiator at 70 °C. After a chain extension reaction with VAc and a basic methanolysis process, a fluorinated amphiphilic polymer was prepared. Based on this, a novel fluorinated polyurethane with flexible fluorinated polymer chains linked to the network was successfully synthesized, which could be used for coating or in some other applications.

## Figures and Tables

**Table 1:** Results of RAFT/MADIX copolymerization of CTFE and BVE.

Sample	Time/h	*Conv.* ^1^	*M_n,th_*	*M_n,NMR_*	*M_n,GPC_*	*M_w_/M_n_*
P1	1	0.11	2500	2400	4600	1.41
P2	2	0.22	5000	5200	6900	1.38
P3	3	0.31	6900	7300	8900	1.43
P4	4	0.40	8900	8500	10,900	1.37
P5	5	0.50	11,000	11,000	13,000	1.37
P6	6	0.56	12,300	12,200	14,500	1.35
P7	9	0.66	14,500	14,800	16,800	1.39

^1^ Conversion of BVE measured by gravimetry.

**Table 2:** Chemical resistance of the fluorinated polyurethane and of poly(CTFE-alt-BVE).

Sample ^1^	Solvent							
	Water	H_2_SO_4_ (10 wt%)	NaOH (10 wt%)	Ethyl Acetate	Acetone	Ethanol	CHCl_3_	THF
FPU	10	10	10	8	8	10	8	8
PCB	10	10	10	0	0	10	2	0

^1^ FPU: fluorinated polyurethane, PCB: poly(CTFE-alt-BVE).

## Supplementary Materials

The following are available online at https://www.mdpi.com/2073-4360/11/9/1440/s1, Figure S1. 1H NMR spectrum of BEDTC recorded in CDCl3 at room temperature; Figure S2. 13C NMR spectrum of BEDTC recorded in CDCl3 at room temperature.
