# Supplementary material for: A New Strategy for the Synthesis of Fluorinated Polyurethane
Source: Polymers (Basel). 2019 Sep 2;11(9):1440. doi: 10.3390/polym11091440 (PMC6780919; doi:10.3390/polym11091440)
Supplement: Supplementary file 1 [file polymers-11-01440-s001.pdf]

Electronic Supplementary Information

A New Strategy for the Synthesis of Fluorinated Polyurethane

Pu-Cheng Wang,[a] Dan Lu,[b] Hu Wang[b] and Ru-Ke Bai[b,]*

[a] Institute of Nuclear Physics and Chemistry, China Academy of Engineering Physics, Mianyang 621000, China.

[b] CAS Key Laboratory of Soft Matter Chemistry, Department of Polymer Science and Engineering, University of Science and Technology of China, Hefei 230026, China.

1. Synthesis of S-benzyl O-ethyl dithiocarbonate (BEDTC)

BEDTC was synthesized according to a previous report.[1] The synthesis was conducted by stirring 40 mL ethanol and 5.6 g KOH (0.10 mol) until a clear solution was formed. Then, 20 mL CS_2 (0.33 mol) was slowly added into the solution, and the mixture was stirred for 10 h at room temperature before excess CS_2 was distilled off at 70 °C. Then, 10 mL benzyl chloride (0.087 mol) in 20 mL of ethanol was added to the residual solution, and the mixture was further stirred at 60 °C for 5 h. After removal of the inorganic salt and most of the ethanol, 50 mL water was added, and the solution was extracted with diethyl ether (3×40 mL). The combined organic layer was dried over anhydrous Na_2SO_4. Removal of the inorganic salt and evaporation of the solvent afforded a yellow oil product: 12.1 g, 65.8%. ^1H and ^{13}C NMR spectra are shown in Figure S1 and Figure S2, respectively.

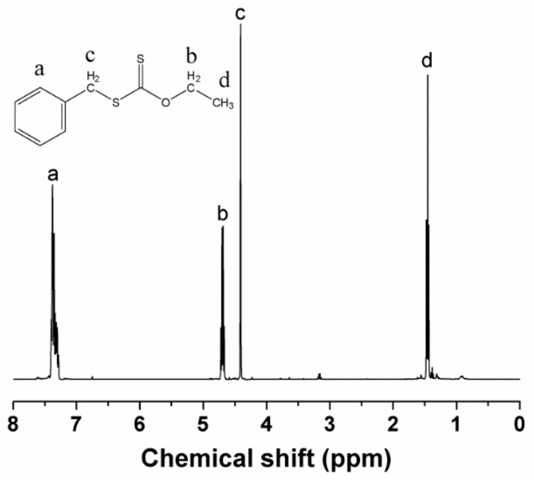

Figure S1. ^1H NMR spectrum of BEDTC recorded in $CDCl_3$ at room temperature.

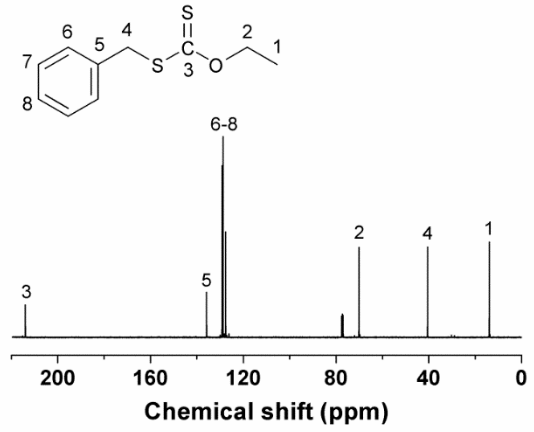

Figure S2. ^{13}C NMR spectrum of BEDTC recorded in $CDCl_3$ at room temperature.

2. Chemical shifts of the copolymers in ^1H NMR spectra

Poly(CTFE-alt-BVE) (δ=ppm): 4.52 (m, 1H), 3.73 (m, 2H), 2.75 (m, 2H), 1.59 (m, 2H), 1.40 (m, 2H), 0.89 (m, 3H).

Poly(CTFE-alt-BVE)-*b*-PVAc (δ=ppm): 4.81 (m, 1H), 4.50 (m, 1H), 3.71 (m, 2H),

2.74 (m, 2H), 1.99 (s, 3H), 1.75 (m, 2H), 1.55 (m, 2H), 1.38 (m, 2H), 0.85 (m, 3H).

Poly(CTFE-alt-BVE)-*b*-PVA (δ=ppm): 4.46 (m, 1H), 3.92 (m, 1H), 3.70 (m, 2H), 2.72 (m, 2H), 1.52 (m, 2H), 1.40 (m, 2H), 1.35 (m, 2H), 0.84 (m, 3H).
